# Fast and Accurate Approaches for Large-Scale, Automated Mapping of Food Diaries on Food Composition Tables

**DOI:** 10.3389/fnut.2018.00038

**Published:** 2018-05-09

**Authors:** Marc Lamarine, Jörg Hager, Wim H. M. Saris, Arne Astrup, Armand Valsesia

**Affiliations:** ^1^Quartzbio SA, Geneva, Switzerland; ^2^Nutrition and Metabolic Health, Nestlé Institute of Health Sciences, Lausanne, Switzerland; ^3^Department of Human Biology, NUTRIM, School of Nutrition and Translational Research in Metabolism, Maastricht University Medical Centre+, Maastricht, Netherlands; ^4^Department of Nutrition, Exercise and Sports, Faculty of Science, University of Copenhagen, Copenhagen, Denmark

**Keywords:** fuzzy matching, food composition tables, food diaries, macronutrient, food mapping, dietary studies

## Abstract

**Aim of Study:** The use of weighed food diaries in nutritional studies provides a powerful method to quantify food and nutrient intakes. Yet, mapping these records onto food composition tables (FCTs) is a challenging, time-consuming and error-prone process. Experts make this effort manually and no automation has been previously proposed. Our study aimed to assess automated approaches to map food items onto FCTs.

**Methods:** We used food diaries (~170,000 records pertaining to 4,200 unique food items) from the DiOGenes randomized clinical trial. We attempted to map these items onto six FCTs available from the EuroFIR resource. Two approaches were tested: the first was based solely on food name similarity (fuzzy matching). The second used a machine learning approach (C5.0 classifier) combining both fuzzy matching and food energy. We tested mapping food items using their original names and also an English-translation. Top matching pairs were reviewed manually to derive performance metrics: precision (the percentage of correctly mapped items) and recall (percentage of mapped items).

**Results:** The simpler approach: fuzzy matching, provided very good performance. Under a relaxed threshold (score > 50%), this approach enabled to remap 99.49% of the items with a precision of 88.75%. With a slightly more stringent threshold (score > 63%), the precision could be significantly improved to 96.81% while keeping a recall rate > 95% (i.e., only 5% of the queried items would not be mapped). The machine learning approach did not lead to any improvements compared to the fuzzy matching. However, it could increase substantially the recall rate for food items without any clear equivalent in the FCTs (+7 and +20% when mapping items using their original or English-translated names). Our approaches have been implemented as R packages and are freely available from GitHub.

**Conclusion:** This study is the first to provide automated approaches for large-scale food item mapping onto FCTs. We demonstrate that both high precision and recall can be achieved. Our solutions can be used with any FCT and do not require any programming background. These methodologies and findings are useful to any small or large nutritional study (observational as well as interventional).

## Introduction

Food composition tables (FCTs) document the nutritional content and properties of food items. These tables are used in conjunction with dietary records, e.g., food diaries, to match consumed food items, and quantify the dietary intake from an individual. The availability of complete and good quality FCTs is required to enable quantitative research in nutritional studies, as well as epidemiological research and public health monitoring. These data are also playing a critical role in studies that aim to monitor an individual's diet and propose personalized recommendations.

Traditionally, FCTs were compiled at a national level with limitations in the data format, depth of annotation and data completeness across different countries. Noticeable efforts have put in placed over the past few years to collect, standardize, and curate FCTs (1–5). In particular, the European Food Information Resource (EuroFIR) has been pivotal in harmonizing data from more 28 national FCTs (including European countries and the USA) (2). Electronically linking these data with food diaries data from observational or interventional studies, provides opportunities to better study the link between Health and Nutrition (6). Yet, dietary consumption is often collected through paper-based food diaries, which requires substantial effort for digitalization (converting records to electronic format) and for food item mapping (for each record, identify its corresponding or closest food item in FCTs and collect the nutritional composition of the matched item). As of today, this effort still remains a manual, expertise-driven exercise. As a direct consequence, such manual mapping is limited to the available study's resources and the retrieved information is limited to a few composition variables (e.g., macronutrients and energy content, and rarely extended to detailed information about micronutrients).

Numerous efforts are being spent in methods for food image recognition using deep learning (7–10) and there is an explosion of mobile applications for food recording. They might prove helpful with future studies by enabling the individual to directly link the consumed food items to a reference database. However these solutions are not intended to solve the mapping issue in studies with existing food records. In addition, their interface, quality of their underlying FCT, and performance still remain to be carefully validated for use in clinical nutrition studies. Hitherto, food item mapping remains a largely un-addressed issue.

Another problem relies in estimating the variability introduced with the mapping. The ideal mapping aims to match the queried food record onto an item from the FCT. In practice, the one-to-one match rarely exists: local FCT may not be extensive enough to enable food matching. Also in multi-centric studies, food items from a specific country would be frequently matched onto a larger FCT from another country that may not have a close equivalent for a specific item from another country. In the absence of a clear on-to-one mapping between a food record and a FCT's food item, several strategies are possible: ignore the food record (thereby introducing missing data), use the closest match, or to create an average profile from several close matches. All three options would introduce variability (or missing data) that needs to be appropriately handled in subsequent statistical analyses. To our knowledge, the variability induced from uncertain food mapping, remains un-addressed in nutritional studies and dietary intakes are analyzed based on the assumption that a perfect match has been found between the food record and one food item from the FCT. This mapping uncertainty is magnified in multi-center studies, where food records from a specific country often need to be translated in English and then mapped onto a English-based FCT [such as the USDA (11) or MW7 (1)]. In this scenario, the English translation may add further to the uncertainty or simply the queried food item may not exist in the English-based FCT.

Finally, variability may also come from the FCTs themselves when they contain different versions of the same food item or when the nutritional content of an item is incorrect [e.g.,when the record was saved using an incorrect unit such as kJ instead of kcal, such erroneous record would stand as an aberrant value (i.e., as an outlier) compared to other similar food items]. Various statistical methods exist to detect outliers. However, outlier detection can only be attempted within a group of coherent, similar items. Also the clustering needs to be made with a granularity that goes beyond the simple food group category. Whilst significant efforts have been spent on data integration and unit harmonization across FCTs, significant efforts remain needed for quality control and data curation. In particular, there is a strong need for metrics to perform food item clustering and subsequently to detect and correct errors in FCTs.

In this study, we attempt automated remapping of a large number of food records (~170,000 individual food records, corresponding to 4,200 distinct food items). The food diary data stemmed from one of the largest weight maintenance dietary intervention of its kind: the Diet, Obesity and Genes study [DiOGenes (12–15)]. Food items were matched to those referenced in the EuroFIR. We define and evaluate an automated approach, based on food name similarity. We also propose an additional approach based on machine learning, to refine mapping of difficult items. Finally, we compared the performance of our approaches, when using the original food name or English-translation.

## Materials and methods

### Ethics

The DiOGenes study (12–15) was performed according to the latest version of the Declaration of Helsinki. Local ethical committees approved of all procedures that involved human participants and written informed consent was obtained from all participants. The present study did not use any clinical data or any individual-level data; only unique food elements (food items defined by their name and macronutrient composition) from food diaries were used.

### Study design and participants

The DiOGenes study is a pan-European, multi-center, randomized controlled dietary intervention program (NCT00390637). The study was conducted in eight European countries: the Netherlands (NL), Denmark (DK), United Kingdom (UK), Greece (GR), Bulgaria (BG), Germany (D), Spain (SP), and Czech Republic (CR). The study has been described in detail previously (12–15). Family eligible for inclusion consisted of at least one overweight (body mass index > 27 kg/m^2^) but otherwise healthy parent aged less than 65 years with at least one healthy child between 5 and 18 years. All eligible adults (*n* = 932) followed a low-caloric diet (LCD) for 8 weeks. The LCD provided 800 kcal per day with the use of a meal-replacement product (Modifast, Nutrition et Santé France). Participants could also eat up to 400 g of vegetables (corresponding to a maximal addition of 200 kcal/day). Families with at least one of the parent achieving at least 8% of weight loss were then included in a 6-month weight maintenance diet (WMD) phase, following either a low/high protein, and glycemic index diet or a control diet.

### Food diary data

Adults completed a 3-day weighed food record for three consecutive days, including two-week days and one weekend day. Records were validated during interview with qualified nutritionists. Food diaries were completed at screening, 2–4 weeks after the randomization in the WMD and 2–4 weeks before completion of the WMD. The participants were instructed to weigh all their foods and to supply information on brand names, cooking and processing. When weighing was not possible, participants were instructed to record the quantity in household measures (cups, glasses, tablespoons). All foods noted in these diaries were coded to foods listed in country-specific FCTs (15). Based on such coding and recorded weight, the macronutrient and energy intake was computed for each record. Additional nutrient information such sugar, starch, fiber, mono- and poly-unsaturated fat was retrieved. In total, 202,000 food records were collected during the study. Yet the retrieved information remained very partial and a high number of missing values were observed (about 7% missing values for the energy content, 17–31% missing values for macronutrient variables; and more than 50% missing values for other variables). In this study, we used data from six centers (NL, DK, UK, GR, BG, and SP) for which an FCT from the same country was available in the EuroFIR. FCT data from Germany and Czech Republic, as provided by EuroFIR, requires additional licenses (to be purchased directly from their respective data source). Thus, in our proof-of-concept study, we focused the mapping onto data available from the standard EuroFIR membership (providing access to FCTs from the same six remaining DiOGenes countries). In total, the food diary records (~170,000) pertained to a unique list of 4,179 food items. For each food item, both original (local-language), and English-translated names were available. At the time of data retrieval (April 2016), the EuroFIR resource provided access to the following databases versions: NL data from the NEVO database version 2014, DK data from the 2009 release, UK from 2008, GR from 2013, BG from 2009, and SP from 2010.

### Statistical analyses

#### Food name comparison using fuzzy matching

**Figure 1:**
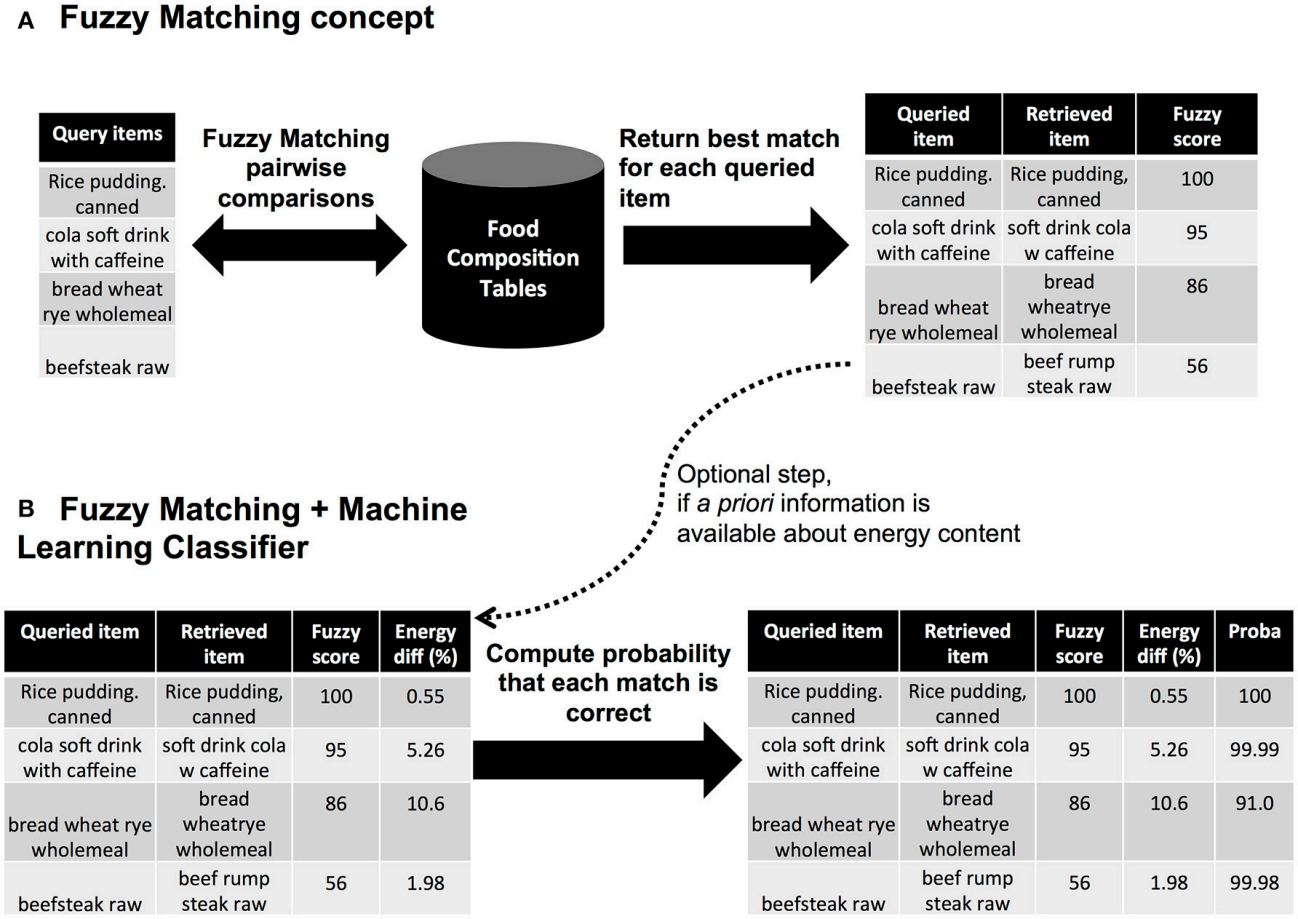
Automated approaches for food item mapping. **(A)** Concept using fuzzy matching, **(B)** Extension of the fuzzy matching using a machine learning classifier (C5.0 trees).

In information theory, comparing two names (strings of characters) is referred to as fuzzy matching and searches a sequence of letters (a string) that matches approximately a pattern. A frequently similarity metric is the Levenstein distance (16) that computes the minimal number of single-characters edits (insertion, deletion, substitution) needed to change one word into another. We used the partial token sort ratio from the FuzzyWuzzyR package (17). This approach is based on the Levenstein distance and computes the ratio of the most similar substring (pattern) between two food names, where each names is split by words (token), and sorted prior the comparison. The resulting score ranges from 0: the two food names are distinct (no common substring is found); up to 100 the two elements are identical (or one of the element is fully included in the second element). Prior computing the fuzzy matching metric, all punctuation marks (commas, semi-columns, points, question-marks, etc.) were removed and all letters set to upper case. Figure 1A summarizes the fuzzy matching concept and provides examples of similarity between food names.

#### Annotation process to review food item matches

To evaluate whether the matches were plausible, the food names (English and original language), the energy and macronutrient content was also investigated. Decision on whether a match DiOGenes-EuroFIR was plausible was defined from a holistic consideration from the food names, the type of process applied to the food (e.g., “boiling” or “frying”), differences in several composition variables (e.g., energy content, fat, sugar content, etc.). In specific case, e.g., when no English translation was available or seemed inaccurate, the two matching elements were reviewed with a Google image search. In case of ambiguity, a conservative approach was used and the match was assigned to as being “non-plausible.”

Upon manual annotation of matches, the overall performance (relevance of mapping; and amount of items that can be mapped) was assessed using the so-called precision-recall curves (18). Specifically, each pair of items (the queried item and its match) has two important attributes: the outcome of the manual review (“plausible match” or “not plausible”) and a single metric that aims to quantify the mapping confidence (e.g., the fuzzy matching score). The assumption is that items with high confidence scores are more likely to be “plausible matches” than items with lower scores. The challenge is to define a threshold on such score and enable automated classification into either plausible or non-plausible match. A precision-recall curve assesses the quality of mapping by investigating a large range of possible thresholds. For each threshold, automated classification can be made for each pair of items and can be contrasted with information from the manual review. This allows deriving the following table:

**Table:** 

		**Status from manual review**	
		**Plausible match**	**Non-plausible match**
**Predicted status**	Predicted plausible match (If score ≥ threshold)	True positives (TP)	False positives (FP)
	Predicted non-plausible match (Score < threshold)	False negatives (FN)	True negatives (TN)

From such table two important metrics can be defined:

- Precision: the number of items correctly classified as plausible matches, divided by the total number of all predicted plausible matches. $\text{From the above table},\;precision=\frac{TP}{TP+FP}$
- Recall: the number of items correctly classified as plausible matches, divided by the total number of all existing plausible matches. $recall=\frac{TP}{TP+FN}$

In a precision-recall curve, each point corresponds to a single threshold and reflects the precision and recall metrics as obtained when using such threshold for automated classification. These curves are useful for two aspects: (1) identify a classification threshold that provides satisfactory precision and recall performance; (2) compares the overall performance from different classification models (with one curve per model).

#### Food item comparison using machine learning

To extend on the fuzzy matching comparison, we defined a machine learning classifier to better distinguish between plausible and non-plausible matches. A machine learning classifier attempts to learn from the data and define a model that can achieve good performance at predicting two classes (e.g., plausible/non-plausible match). Numerous approaches exist in the field of machine learning. Here, we used a C5.0 classification tree. C5.0 models are one type of classification trees and are extremely popular (19, 20). A classification tree defines a decision process, where each node in the tree is a test on an input variable and results in a binary decision forming two sub-nodes (sub-groups). Each sub-group is then tested with another test until a final decision can be made. In our analyses, the final decision corresponds to whether a given pair of items corresponds to a plausible match. Classification trees have the advantage to be easily interpretable compared to other more complex models (e.g., neural networks). A C5.0 model aims to test the most informative variables first and to define a binary split that optimize the similarity of the resulting sub-groups. In more details, a C5.0 model is based on the concept of information entropy (a measure of the homogeneity within a group) and extracts informative patterns from the data to achieve a binary classification. Each node of the tree is built by defining a binary rule based on the variable that provides the maximal information gain (by defining the most homogeneous sub-groups). Each resulting node is then split again until no more splits are possible. This type of models is robust in the presence of missing data and the resulting classification rules are easily interpretable. In addition, the performance of classification can be significantly improved by using a boosting strategy (21). Boosting enables to define several models (some that may only have a moderate performance) and to combine them into a better, consensus meta-model.

Our C5.0 classification trees used as input variables the fuzzy matching score and the percentage of difference in energy content between the two food items. To avoid biases when comparing energy content, the nutritional content from all food items (incl. DiOGenes and EuroFIR items) were scaled to 100 g portions. The percentage of difference in energy content was computed as follows:

$$\begin{array}{l} Ediff=100*abs\\ \left(\frac{Diogenes\text{\_}item\text{\_}energy\text{\_}content-EuroFIR\text{\_}item\text{\_}energy\text{\_}content}{Diogenes\text{\_}item\text{\_}energy\text{\_}content}\right) \end{array}$$

We built the C5.0 model using the following approach. First, we used a 20% random subset of the EuroFIR resource and computed all pairwise comparison with the DiOGenes food items. Next, we restricted the list of all pairs (n > 3,300,000) to those with either:

- fuzzy matching score > 75% and absolute difference in energy content < 25%
- fuzzy matching score > 90%

This smaller list (*n* = 2,625 pairs) was then manually reviewed to indicate in a new column whether the match was plausible (see above section on annotation process). From the list of annotated matches, we then built a boosted C5.0 classification tree using the C50 R package (22). Two models were trained, one based on a comparison between original food names and another one based on a comparison using the English-translated food names. The resulting trees are shown in Supplementary Figure 1. These models allowed for a new pair of item to derive the probability that the match is correct, given their similarity in term of fuzzy matching and energy content (see illustration in Figure 1B). For simplicity in the manuscript, we refer to these outputs as the C5.0 probabilities. These probabilities were obtained using the *predict* function from C50 R package.

#### Code availability

Our code is available from R packages released under General Public Licenses (GPL licenses). These packages enable to perform fuzzy matching comparisons, irrespectively of any FCT [FoodMapping package released under GPL version 2 (GPL-2); available from https://github.com/armandvalsesia/Foodmapping]; and also to compute the probability that a match is correct based on pre-trained C5.0 models [FoodC5 package released under GPL-3, available from https://github.com/armandvalsesia/FoodC5]. The code is optimized for a large number of comparisons and does not require access to high performance computers or clusters. Documentation and quick-start tutorial are also available.

## Results

### Fuzzy matching concept

Nutritionists encoding food diaries initiate their searches by searching the food name in a FCT interface. Then, from a list of elements containing the queried name or keywords, the nutritionist would decide which element is the closest match. When the returned elements have identical or near-identical names compared to the queried element, the decision could be automated thereby reducing the number of items that requires an expert decision. We thus sought to investigate possible approaches to map food items from the DiOGenes study onto the EuroFIR FCTs.

**Figure 2:**
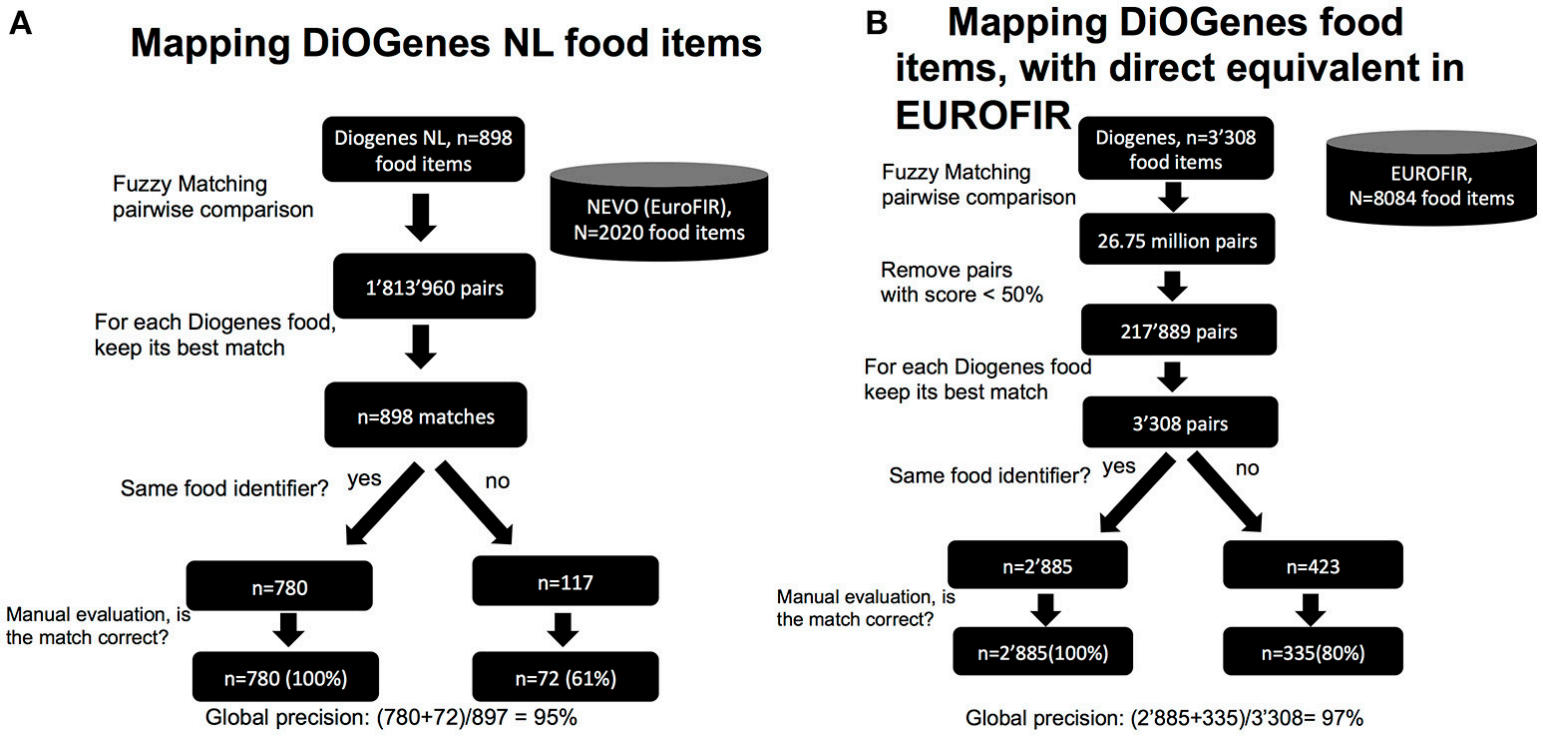
Proof of concept: performance at mapping existing items onto FCTs. **(A)** Example with DiOGenes NL items mapped onto NEVO; **(B)** Example with all DiOGenes items mapped onto EuroFIR.

We first assessed mapping items from one single country and for which the reference FCT is still available. During the DiOGenes study, food diaries from the Netherlands (NL) were mapped onto a release of Dutch Food Composition Database [the NEVO database (23)]. NEVO has evolved and is still maintained. It is available from the Dutch national institute for public health and environment and has been integrated in the EuroFIR. EuroFIR keeps a unique food identifier code for each food item per country and keeps reference to the original (FCT) food identifier code. In the DiOGenes food data, food identifier codes were also recorded and we were able to cross-match all 898 DiOGenes NEVO items onto the current NEVO release. Manual investigation showed that all these matches were correct. This mapping constitutes a very valuable resource to assess the performance of an automated approach, notably by assessing how many of these known matches could be retrieved. We thus applied the fuzzy matching approach described in Figure 2A. Out of 898 food items, 780 (87%) of the mapped items (using the fuzzy matching) shared the same food identifier codes, indicating that the reference item was found. The remaining fraction (117 items) was investigated manually. We found that 72 of those (61%) were plausible matches. Thus, our automated approach was able to correctly remap 852/898 (95%) of the queried DiOGenes NL food items.

Next, we applied a similar strategy to 4,179 DiOGenes food items from six countries (NL, DK, UK, GR, BG, and SP) that have an FCT from the same country in EuroFIR. Among those items, 3,308 (80%) could be cross-matched using their food identifier codes; indicating that they had a clear equivalent. Using the fuzzy matching approach, we were able to remap all 3,308 items, with a global precision equals to 97% (Figure 2B). These results demonstrate that when a queried food item is already present in the FCT, our approach would find it.

### Real-world, large-scale example: application to all food items

The proof of concept focused on items that could be matched by food identifier code, and thus are assumed to have an equivalent in EuroFIR. However, in a real-world problem, the fraction of items that is already present in the FCT is unknown. Also for items without a direct equivalent, it is unknown whether those could be mapped onto a similar item.

We thus sought to apply the fuzzy matching approach to the items that could not be cross-matched based on their food identifier codes. This corresponded to 871/4179 (20%) of the DiOGenes food items. By applying the same process (i.e., find the best match) and upon manual review of the hits, we obtained annotated results: each pair of matching items was annotated as being a plausible match or an incorrect one. This annotation enabled to derive specific performance metrics, for different thresholds on the fuzzy matching score. For example, the precision (percentage of correctly mapped items) can potentially be improved by considering pair of items for which the fuzzy score is greater than a given threshold (e.g., 90% instead of 50%). However, increasing such stringency would *de facto* limits the number of queried items that can be mapped. Estimating the recall rate (percentage of mapped items) is another important indicator of performance.

**Figure 3:**
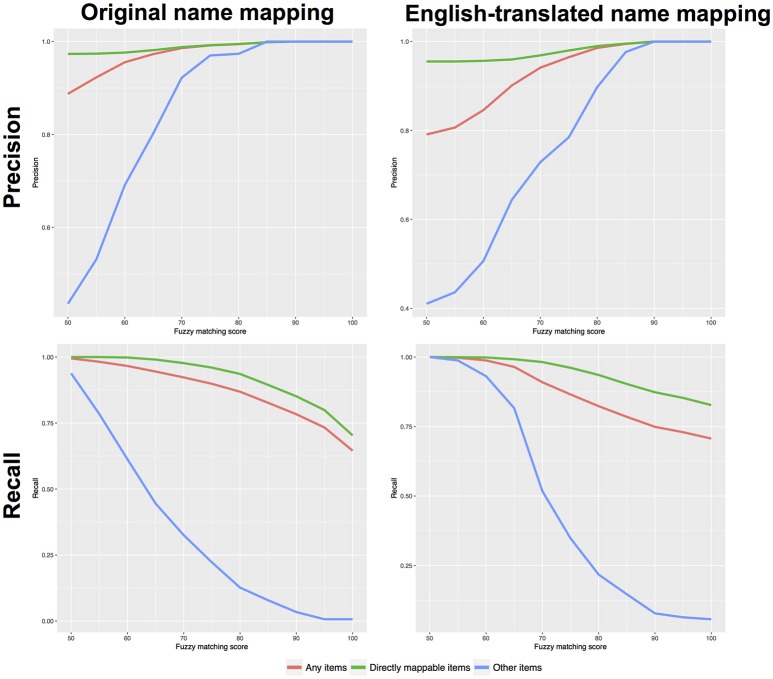
Precision and recall curves, as a function of fuzzy matching scores. Left plots correspond to a mapping using the original food names; right plots correspond to mapping food names using their English translation.

Figure 3 presents the performance (precision and recall) as a function of increasing fuzzy score thresholds. Two approaches were tested: a mapping using the original country food names (e.g., Danish name) and using the English-translated names. For each approach, Figure 3 presents the performance for items that were previously cross-matched based on their food identifier codes (“directly mappable”); items that could not be cross-matched (“other items”); and both type of items (“all items”).

As expected, the directly mappable items (green curves) achieves very good performance, already with a permissive fuzzy score filter (with a 50% fuzzy score threshold) all items could be mapped (recall = 100%) and the precision is 97%. By contrast, the precision for the more difficult items (“other items”) was not good and only achieved 43.6% (when using the original food name). Similar precision was achieved when using the English-translated name. Therefore, for such difficult items, more stringent thresholds are required. Using thresholds at 70% increased the precision to 92.2 and 73%, respectively for the mapping with Original and English-translated names. However, the recall rates were reduced to 32.5% and 51.8%. The “all items” group is representative of a real-world mapping and 20% of these items are the difficult ones. Here, the global performance was found acceptable with permissive fuzzy score thresholds. At 50% threshold, the precisions were 88.7 and 79.1%, respectively for the Original and English-translated name mapping. For both mapping, the recall rates were > 99%. Using a threshold at 70%, the performance can be significantly improved with precision > 94% and recall > 91%.

From Figure 3, specific fuzzy score thresholds can be derived to achieve high precision (e.g., 75%). Table 1 illustrates how a desired precision (e.g., 75 or 80%) would influence the fuzzy score thresholds. For e.g., when mapping items using their original name, a threshold at fuzzy score > 50% would enable more than 75% precision when mapping easy (“directly mappable”) items while a threshold > 63% would be required for difficult items (those without direct equivalent). Therefore, to enable good precision for all items, the stringent threshold > 63% should be used. With such threshold, the precision for all items would be close 97% with a recall rate > 95% (Table 2).

**Table 1:** Thresholds required to achieve 75 or 80% precision, with the fuzzy matching approach.

		**Matching with original name**			**Matching with English-translated name**		
**Target**	**Item type**	**Threshold**	**Precision (%)**	**Recall (%)**	**Threshold**	**Precision (%)**	**Recall (%)**
precision > = 75%	Any	50	88.75	99.49	50	79.11	100.00
precision > = 75%	directly mappable	50	97.34	100.00	50	95.53	100.00
precision > = 75%	Others	63	76.84	50.00	75	78.49	34.81
precision > = 80%	Any	50	88.75	99.49	50	79.11	100.00
precision > = 80%	directly mappable	50	97.34	100.00	50	95.53	100.00
precision > = 80%	Others	65	80.25	44.52	80	89.78	21.73

**Table 2:** Threshold required to achieved 75% precision for all three-item classes (“Any,” “Mappable,” “Other”), with the fuzzy matching approach.

**Threshold**	**Food name comparison**	**Any items**	**Directly mappable items**	**Other items**
63	Original name	PR = 96.81, Rec = 95.19	PR = 97.98, Rec = 99.29	PR = 76.84, Rec = 50
75	English-translated name	PR = 96.49, Rec = 86.55	PR = 98, Rec = 96.14	PR = 78.49, Rec = 34.81

When mapping items using their English translation, different threshold should be used. For precision > 75%, the fuzzy score threshold should be > 75% (Table 1). This will enable a precision close to 96.5% for all items, with a recall rate equal to 86.5% (Table 2).

### A machine learning approach to refine food item matching

As expected, using stringent thresholds to increase the precision leads to smaller recall rates. In particular, at precision > 75%, the recall rate for difficult items is less than 50% (Table 1). Since difficult items only represent 20% of all queried food items, it means that only 10% of those cannot be mapped automatically and would require an expert-driven matching. This constitutes an improvement over the current situation (where all items are matched manually). Still, we sought to explore additional approaches to improve such matching.

Previous results showed that mapping food names using their English translation would not improve the recall compared to a mapping using the original name (recall rates were 34.8 vs. 50%, respectively). Therefore, additional data would be needed to improve the matching. We rationalized that such information should be easy to acquire. Inherently, if extensive information were already known about the macro- and micro-nutrient composition from a queried food item, it would mean that the mapping was already done once. Instead, we sought to use *a priori* information about the food content that could be easy to acquire. We made the assumption that some rough estimates about the total energy content could be obtained during the digitalization phase (i.e., converting paper-based food diaries to electronic diaries). Such estimates would be used to compute the difference in energy content between the queried and retrieved food items; which could potentially be useful to discriminate whether the match is plausible or not.

To assess this approach, we trained C5.0 classification trees (see section Materials and Methods). We observed that this approach had some potential to map items that could not be mapped with the fuzzy matching alone. For e.g., when searching for “beefsteak raw,” the top matching item would be “beef rump steak raw.” However the corresponding fuzzy score would only be 56% and may not pass stringent fuzzy score filters. Yet, the difference in energy content is relatively low between these two items (< 2%) and thus the probability that the match is correct is very high (99.98%). Conversely, this approach could help discriminate between highly similar food names but that pertain to very different food products. “hake raw” and “hare raw” differ only by one letter and thus the resulting fuzzy score would be high (88%). Yet, those two items differ by 25% in term of energy content. With our C5.0 approach, the resulting probability that the match is correct would be relatively low (52%, i.e., close to a random guess).

### Large-scale performance of the machine learning approach and comparison with the fuzzy matching approach

**Figure 4:**
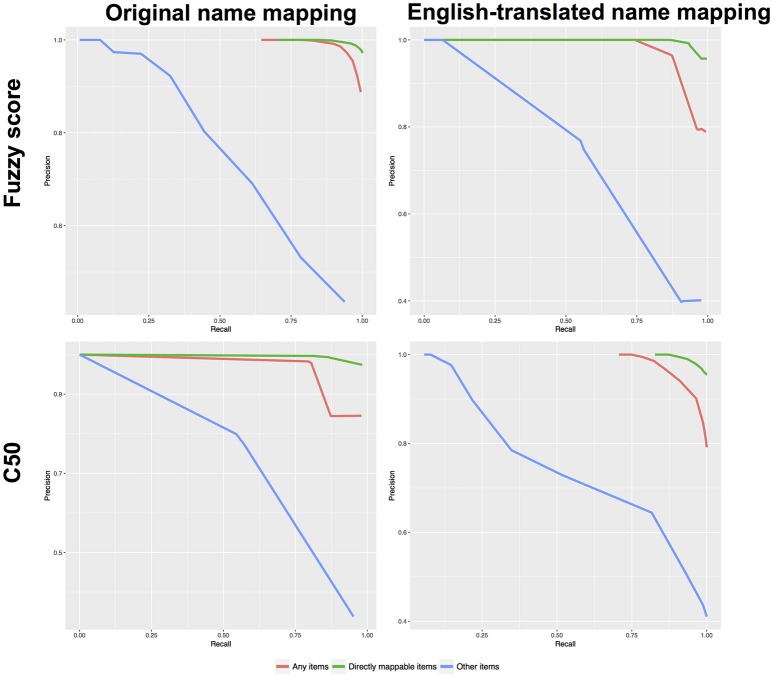
Precision-recall curves for the two approaches (fuzzy matching, machine learning) and using either original or English-translated food names.

We next evaluated the performance from our machine learning approach and compared it to previous results using only the fuzzy scores. Table 3 shows the required thresholds to achieve 75 or 85% precision and Table 4 provides the performance when using a single threshold that achieves at least 75% precision for all item categories. The performance (precision vs. recall) for all our approaches (fuzzy scores/C5.0 combined with either original- or English-translated food name mapping) is shown in Figure 4. When mapping the “Any items” list, all approaches had very good performance (strong precision and recall). With relaxed thresholds (fuzzy score > 50% or C5.0 probability > 50%), all four approaches led to comparable performance with precision > 78% and recall > 97%.

**Table 3:** Thresholds required to achieve 75 or 80% precision, with the machine learning approach.

		**Matching with original name**			**Matching with English-translated name**		
**Target**	**Item type**	**Threshold**	**Precision (%)**	**Recall (%)**	**Threshold**	**Precision (%)**	**Recall (%)**
precision > = 75%	Any	50	84.55	97.98	50	78.86	99.34
precision > = 75%	directly mappable	50	97.41	98.23	50	95.60	99.64
precision > = 75%	Others	85	77.31	57.19	84	76.85	55.12
precision > = 80%	Any	50	84.55	97.98	64	79.53	97.87
precision > = 80%	directly mappable	50	97.41	98.23	50	95.60	99.64
precision > = 80%	Others	92	79.90	54.45	92	100.00	6.54

**Table 4:** Threshold required to achieved 75% precision for all three-item classes (“Any,” “Mappable,” “Other”), with the machine learning approach.

**Threshold**	**Food name comparison**	**Any items**	**Directly mappable items**	**Other items**
85	Original name	PR = 97.89%, Rec = 80.5%	PR = 99.55%, Rec = 82.61%	PR = 77.31%, Rec = 57.19%
84	English-translated name	PR = 96.46%, Rec = 87.32%	PR = 99.23%, Rec = 93.29%	PR = 76.85%, Rec = 55.12%

The performance can be further decomposed to distinguish between “easy cases” (those that exists in the FCTs; i.e., “directly mappable”) and “difficult cases” (without an equivalent). For easy cases, there was no improvement with the machine learning approach compared to the fuzzy matching approach. In fact, the fuzzy matching approach had better precision rate. However, for difficult cases, the machine learning approach improved significantly the recall rate, while keeping a comparable precision. With thresholds to ensure > 75% precision, the recall rate increases from 50 to 57% when mapping items with their Original name. When mapping items using their English-translated names, the recall rate improves from 34.8 to 55.1%.

## Discussion

In this study, we explored computational approaches to automate food item mapping onto FCTs. Up to date, noticeable emphasis had been placed to collect FCT data; to enable some level of harmonization across FCT and facilitate data access through databases and user-interfaces (1–5). Yet, this did not address the problem to automatically map food records.

We found that the simpler approach: fuzzy matching, provided very good performance. Under a relaxed threshold (fuzzy score > 50%), this approach enabled to remap 99.49% of the queried items with a precision equal to 88.75%. With a slightly more stringent threshold (fuzzy score > 63%), the precision could be significantly improved to 96.81% while keeping a recall rate > 95% (i.e., only 5% of the queried items would not be mapped). The more complex approach (based on a C5.0 classifier) enabled to increase the recall rate of difficult items and could potentially be used for items that cannot be mapped with fuzzy matching.

In this study, we mapped the DiOGenes food items using six FCTs available from the EuroFIR resource. However, our approach and code implementation is FCT-agnostic and can be used with any other data source. This provides flexibility to use many different FCTs together and to use any customized/private FCT. Also the starting point is only food names, which makes the approach easily applicable to any other nutritional study and other type of dietary assessment methods.

All the code required to perform fuzzy matching and compute the C5.0 probabilities is freely available as open source R packages (available from GitHub). Our implementation is optimized to enable large number of comparisons: on a standard laptop (with a 2.3 GHz Intel Core i7 processor), 100,000 pair-wise comparisons take < 20 s and 1,000,000 comparisons take 205 sec (~3.5 min). Significant speed improvements can be made using parallel computing (either with multi-threading or using distributed computing on a grid). Quick-start tutorials and documentation are available; and the code can be used with a very basic knowledge of the R language.

Traditionally, food mapping was performed manually with each single item to be queried against a reference FCT. Then the expert would need to sift through the list of retrieved items, identify the most relevant match and somehow export the required information (e.g., either a food id code or directly the available nutrient composition). Such process is time-consuming; in our experience with the DiOGenes study and other dietary interventions, it takes on average 5 min per item (with a range between 3 and 8 min, depending on the item complexity and the nutritionist's familiarity with the food item). By contrast, our approach enables to fully automate the mapping and can be completed within a few minutes for over a million comparisons without the need for a human intervention.

Such fast and deterministic process enables to rerun the mapping with newest releases of FCTs and to acquire additional information on the nutrient composition. For e.g., upon the initial manual DiOGenes food item mapping, nutrient composition was retrieved for macronutrients and 13 nutrient variables. Using our automated approach enabled to retrieve all nutrient composition variables as available from the queried FCT (for e.g., an automated mapping onto NEVO would retrieve more than 128 composition variables). On average over the six DiOGenes centers, an additional list of 20 nutrients was added to the food records information. Significant improvement was also reached for the amount of missing values. The initial records had 17–31% missing values for macronutrient variables and more than 50% missing values for other variables. Upon automated remapping, these percentages were reduced below 2.3% for macronutrients and below 10% for other variables (except for alcohol content whose percentage of missing values and strictly zero values was reduced from 98 to 91%).

An additional benefit of the automated approach is the ability to quantify mapping uncertainty. With a manual mapping, the uncertainty cannot easily be quantified by objective means (if at all) and is typically not captured. By contrast, the automated approach computes a mapping confidence metric (similarity or probability), which can be used for ad-hoc post-filtering and could also be taken into account in subsequent statistical analyses.

Throughout our food item review, we observed variability between different versions of the same food item. For e.g., 100 gr portion of raw garlic would be recorded with an energy content varying between 305 and 670 kcal. We did not observe food items recorded with incorrect units for energy composition (kJ instead of kcal). Yet with such volume of information, data curation (including detection and correction of errors) remains a challenge and a thorough review of each composition variables cannot be performed without automated approaches. Specifically, automated outlier detection (in food nutrient values) would help curation and provide new tools for quality control. However, outlier detection can only be performed when similar items are grouped together. While FCTs provide a food group label that could help pre-cluster food items, this information remains very incomplete. In our EuroFIR subset, about 25% of the food items have no food group information. Our fuzzy matching approach could be of help with such issue. It can be used as similarity metric and would enable to cluster similar items together. Then from such clusters, the individual composition variables can be assessed to identify potential outliers. Such approach would help improving further the quality and completeness from FCTs.

Current efforts in data integration and harmonization across FCTs (1–5) focus on renaming nutrient composition variables using a unified nomenclature and deriving the composition values using consistent units. While this is necessary for combining FCTs in a consistent manner, it does not solve the missing value issue. This situation exists within a same FCT where the different food items would inherently have missing values for one or more composition variables (nutrients). This problem is magnified when combining data across different FCTs that have different number of nutrients. For e.g., NEVO is by far the richest database (with information for 128 different nutrients) whilst other FCTs provide information for macronutrients and a few micronutrients. While there is some guidance on how to estimate missing nutrient values (24), it is a manual, expertise-driven, decision and the literature remains scarce for imputation of missing values in FCTs using computational approaches. There is some guidance for recipe calculation however that would not solve the issue at the ingredient-level (25). Our fuzzy matching approach could be used to cluster together similar food items (independently whether they are cooked food or single ingredients) and could potentially prove useful to impute the missing values (using a strategy similar to traditional k-nearest neighbors). Such imputation process could also be improved by using a composite measure of similarity based on both fuzzy scores and similarity in term of the available nutrient composition. Food item clustering based on fuzzy matching also opens new possibilities with respect to FCT data integration. It would enable to keep track of possible modifications between different versions of the same FCT. Finally, with the availability of FCTs from different countries, such clustering would enable to reduce redundancy across different FCTs and to derive a single, more comprehensive meta-FCT.

In summary, we propose strategies to perform food item mapping at large-scale. Our extensive benchmark demonstrates that both high precision and recall can be achieved. Previously food mapping was a manual, time-consuming and expertise-driven process. These new tools provide a powerful alternative to clinicians and nutritionists, who were performing manually these tasks. In addition to reducing significantly the burden and saving time, it makes the process fully reproducible allowing going back to specific matches in a deterministic manner.

To the best of our knowledge, this is the first time that automated solutions are proposed. These methodologies and findings are useful to any nutritional study (observational as well as interventional) and can be applied in both small and large data collections.

## Author contributions

AV: Conceived, designed, and supervised the present study; WS and AA: Designed and supervised the DiOGenes clinical trial; JH contributed data; AV: Developed the statistical approach and the proof of concept; ML: Implemented the approach and optimized R code implementation; AV and ML: Analyzed and interpreted the data; AV: Wrote the paper with input from all authors. All authors read and approved the paper. AV had primary responsibility for final content.

### Conflict of interest statement

AV and JH are full-time employees at Nestlé Institute of Health Sciences SA. ML is a full-time employee at QuartzBio SA. WS reports having received research support from several food companies such as Nestlé, DSM, Unilever, Nutrition et Sante and Danone as well as Pharmaceutical companies such as GSK, Novartis and Novo Nordisk. He is an unpaid scientific officer for the International Life Science Institute, ILSI Europe and reports personal fees from Cosun NL. AA reports personal fees from Crossfit, USA, during the conducts of the study; personal fees from Dutch Beer Institute, NL, Feast Kitchen A/S, Denmark, Groupe Éthique et Santé, France, McCain Foods Limited, USA, Nestlé Research Center, Switzerland, Weight Watchers, USA, BioCare Copenhagen, Zaluvida, Switzerland, Basic Research, USA, Beachbody, USA, Danish Agriculture & Food Council, Novo Nordisk, Denmark, Pfizer, Germany, Saniona, Denmark, Sanofi-Aventis, Germany, S-Biotek, Denmark, Scandinavian Airlines System, Denmark, and from TetraPak, Sweden; personal fees and other from Gelesis, USA; grants from Arla Foods, DK, Danish Dairy Research Council, and Gelesis, USA outside the submitted work. In addition, AA has a patent pending to the University of Copenhagen Methods of inducing weight loss, treating obesity and preventing weight gain. (Licensee Gelesis, USA) and Biomarkers for predicting degree of weight loss (licensee Nestec SA, CH), and he is co-inventor of a number of other patents owned by the University in accordance with Danish law. AA receives royalties for the books “Verdens Bedste Kur”/Politikens Forlag, Denmark, 2012 (subsequently published in English as “World's Best Diet”/Penguin, Australia, and “The Nordic Way”/Random House, USA), and “Spis dig slank efter dit blodsukker” (Eat according to your blood sugar and be slim)/Politikens Forlag, Denmark, 2017. Co-author of several books in the pipeline about personalized nutrition for weight loss. Co-owner and member of the Board of the consultancy company Dentacom Aps, Denmark, co-founder and co-owner of UCPH spin-outs Mobile Fitness A/S & Flaxslim ApS (where also member of Board, 2015-), & Personalized Weight Management Research Consortium ApS (Gluco-diet.dk /2017-).
