# Supplementary material for: Fast and Accurate Approaches for Large-Scale, Automated Mapping of Food Diaries on Food Composition Tables
Source: Front Nutr. 2018 May 9;5:38. doi: 10.3389/fnut.2018.00038 (PMC5954085; doi:10.3389/fnut.2018.00038)
Supplement: Supplementary file 1 [file Image_1.PDF]

Supplementary Figure 1 C5.0 classification trees using either English or Original food name for fuzzy matching

A: Initial C5.0 tree using English food name   B: Initial C5.0 tree using original food name

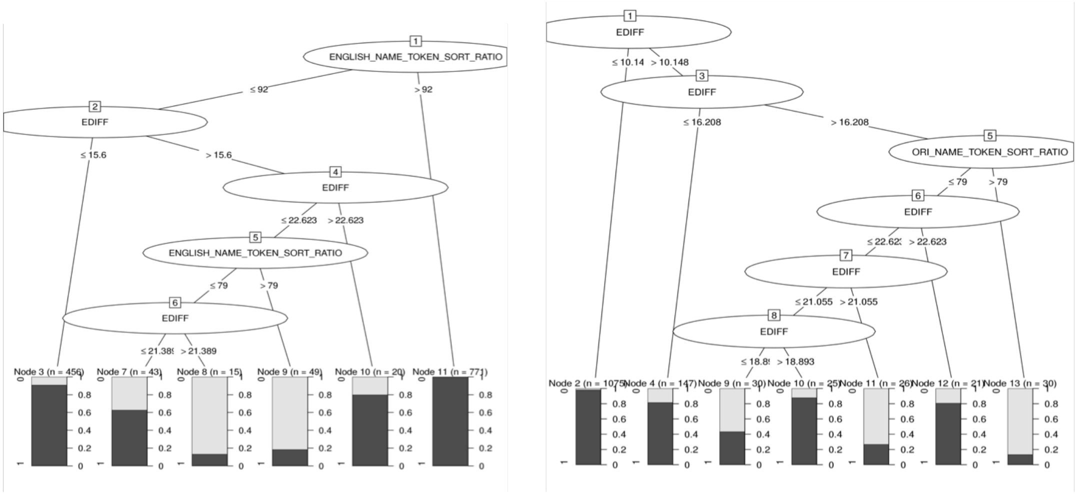

The classification errors are 5.3% and 3%, respectively for a mapping with the English-translated food name, or with the original food name. In these trees, EDIFF corresponds to the percentage of energy content difference between the two matched items; the TOKEN_SORT_RATIO is the fuzzy score (food name similarity).
